# Dead space in acute respiratory distress syndrome: more than a feeling!

**DOI:** 10.1186/s13054-016-1381-7

**Published:** 2016-07-30

**Authors:** Lluis Blanch, Josefina López-Aguilar, Umberto Lucangelo

**Affiliations:** 1 Critical Care Center, Hospital de Sabadell, Corporació Sanitària Universitària ParcTaulí, Universitat Autònoma de Barcelona, c. ParcTaulí 1, 08208 Sabadell, Spain; 2 CIBER de Enfermedades Respiratorias, Instituto de Salud Carlos III, Madrid, Spain; 3 Department of Perioperative Medicine, Intensive Care and Emergency, Cattinara Hospital, Trieste University, Trieste, Italy

Why clinicians are slow to implement advances in diagnosis and treatment from well-designed clinical trials is a continuously debated question in critical care. For instance, prone positioning significantly improves mortality in patients with severe acute respiratory distress syndrome (ARDS), but the usefulness of recruitment measures in this population is still under debate. Nevertheless, a recent observational study in intensive care units in 50 countries found that prone positioning was used in only 16.3 % of patients with severe ARDS, whereas recruitment maneuvers were used in 32.7 % [1]. Similarly, despite the established usefulness of measuring physiologic variables such as dead space in mechanically ventilated ARDS patients, this practice is not widely employed.

Dead space refers to lung areas that are ventilated but not perfused. Dead space comprises two separate components: airway dead space (the volume of areas that do not contribute to gas exchange) and alveolar dead space (the volume of well-ventilated alveoli that receive minimal blood flow). The physiologic ventilatory dead space fraction (VD/VTphys) is usually defined as the fraction of tidal volume (VT) that does not participate in gas exchange [2, 3]. Currently, dead space is measured at the bedside by volumetric capnography, which reports expired CO_2_ elimination as a function of expired VT, and VD/VTphys is calculated using the Enghoff’s modification of Bohr’s original equation: VD/VTphys = (PaCO_2_ – PECO_2_)/PaCO_2_, where PaCO_2_ is the arterial partial pressure of CO_2_ obtained by arterial blood sampling and PECO_2_ is an estimate of mixed expired partial pressure of CO_2_ obtained from the mid-portion of phase III of the volumetric capnogram [2, 3]. Modern volumetric capnographs incorporate this physiologic approach, enabling intensivists to measure VD/VTphys at the bedside. However, if data generated breath-by-breath by capnographs are not integrated and analyzed together with data coming from other physiologic monitors and lung image analysis, their clinical meaning could be incomplete and even misleading.

Over 40 years ago, Suter et al. [4] pointed out that increasing positive end-expiratory pressure (PEEP) in ARDS augments blood oxygenation and decreases shunt. Although they used the maximum level of oxygen transport to determine the optimum levels of PEEP, they showed that maximizing total compliance and minimizing physiologic dead space (VD/VTphys) yielded the best results. Interestingly, the decrease in VD/VTphys occurred with no significant change in anatomic dead space. The authors state that “this observation supports the concept of the recruitment of previously atelectatic lung areas leading to an increase in compliance and a decrease in alveolar dead space, whereas overdistension of alveoli decreases compliance and increases alveolar dead space” [4]. This is, in a nutshell, exactly what intensivists strive for at the bedside: to recruit the lung without doing harm. Unfortunately, in critical care practice dead space is not routinely measured at the bedside. Factors that explain this reluctance to monitor dead space at the bedside include difficulties in understanding physiologically derived information and in interpreting capnograms, together with the lack of integration of CO_2_ waveforms and derived data with other respiratory measurements.

There are several reasons why dead space is an attractive parameter that should be routinely monitored in critical care. First, dead space has important prognostic implications. In ARDS, alveolar and endothelial cell injuries result in alterations of the pulmonary microcirculation in all lung compartments with high and low ventilation/perfusion (V/Q) ratios. Since VD/VTphys reflects alterations in V/Q ratios, it is affected by any type of V/Q mismatch. Although the Enghoff approach to calculating VD/VTphys can be contaminated by the large shunt fractions present in ARDS, the result is a good global index of the efficiency of the gas exchange of a lung [3, 4]. Nuckton et al. [5] demonstrated that a high VD/VTphys was independently associated with an increased risk of death in patients with ARDS. Other groups found similar results in early and intermediate phases of ARDS [6] and in patients with ARDS according to the Berlin definition undergoing lung-protective ventilation [7]. Moreover, during the first 2 days of ARDS, the evolution of noninvasive capnographic indices such as the ratio between alveolar ejection volume and VT, which can be calculated without arterial blood gas sampling, has a prognostic value similar to VD/VTphys [8]. Finally, Siddiki et al. [9] found that VD/VTphys measurement using CO_2_ production estimated from the Harris-Benedict equation predicted mortality in patients with acute lung injury (ALI) in a dose-response manner.

Whether VD/VTphys is useful for PEEP titration is a matter of debate. In ARDS, ideal PEEP titration achieves a balance between maintaining optimal alveolar recruitment and reasonably avoiding lung overdistension. In some studies in patients with ARDS, adequate PEEP corresponded to the lowest VD/VTphys [10] and to the lowest arterial to end-tidal PCO_2_ gradient [11]; PEEP levels also correlated well with the extent of quantitatively measured lung inhomogeneities assessed on computed tomography (CT) images of the lung [12]. Other studies, however, found no effect [13]. This discrepancy could occur due to the different effect of PEEP in patients with various degrees of lung injury or, in positive PEEP responders, the reduction in alveolar dead space compensated for the concurrent increase in airway dead space [13, 14]. Experimental studies clearly suggest that dead-space variables, in particular the ratio of alveolar dead space to VT and the gradient between arterial and end-tidal CO_2_, might become a useful bedside tool for implementing a lung protective ventilation strategy in the context of recruitment and a PEEP titration procedure [14]. In the clinical setting, variations in PaCO_2_ in conditions of unchanged CO_2_ production, respiratory rate, and VT might be a surrogate of variations in dead space. Gattinoni et al. [15] found increased 28-day survival in ALI/ARDS patients in whom prone positioning reduced PaCO_2_. Prone positioning probably improved the efficiency of alveolar ventilation by decreasing VD/VTphys. By contrast, in patients who do not respond to recruitment maneuvers, decreased alveolar ventilation and increased PaCO_2_ probably reflect a worsening of lung injury.

In summary, volumetric capnography could provide powerful breath-by-breath physiologic information about the efficiency of alveolar ventilation and perfusion in patients with ARDS. Improved efficiency of alveolar ventilation is an important marker of patients who will survive ARDS, and can be determined at the bedside by a decreased ratio between physiologic or alveolar dead space and VT.

## Abbreviations

ALI, acute lung injury; ARDS, acute respiratory distress syndrome; PaCO_2_, arterial partial pressure of CO_2_; PECO_2_, expired partial pressure of CO_2_; PEEP, positive end-expiratory pressure; V/Q, ventilation/perfusion; VD/VTphys, physiologic ventilatory dead space fraction; VT, tidal volume
